# Evaluation of COVID-19 policy efficiency in 27 European OECD countries: a data envelopment analysis

**DOI:** 10.1186/s12913-026-14508-z

**Published:** 2026-04-11

**Authors:** Xiaoyu Tang, Yuhan Lou, Sun Sun, Mevludin Memedi, Ayako Hiyoshi, Scott Montgomery, Wenhua Tian, Yang Cao

**Affiliations:** 1 Clinical Epidemiology and Biostatistics, School of Medical Sciences, Faculty of Medicine and Health, Örebro University, Örebro, 70182 Sweden; 2 School of Social Development and Public Policy, Fudan University, Shanghai, 200433 China; 3 Department of Epidemiology and Global Health, Umeå University, Umeå, 90187 Sweden; 4 College of Business, Alfaisal University, Riyadh, 11533 Saudi Arabia; 5 Centre for Empirical Research on Information Systems, Örebro University School of Business, Örebro, 70182 Sweden; 6 Clinical Epidemiology Division, Department of Medicine, Karolinska Institutet, Solna, Stockholm, 17177 Sweden; 7 Department of Epidemiology and Public Health, University College London, London, WC1E 7HB UK; 8 Unit of Integrative Epidemiology, Institute of Environmental Medicine, Karolinska Institutet, Stockholm, 17177 Sweden

**Keywords:** COVID-19 pandemic, Health policy, Efficiency, Data envelopment analysis, Evidence-based evaluation

## Abstract

**Background:**

The COVID-19 pandemic compelled governments worldwide to adopt diverse public health and economic policies. However, the relative efficiency of these interventions across European countries has not been comprehensively evaluated. This study assessed the efficiency of COVID-19 policy responses in 27 European countries of the Organization for Economic Co-operation and Development (OECD), with the aim of identifying factors associated with effective pandemic management. The time period of this study is from January 1, 2020 to December 31, 2022.

**Methods:**

A data envelopment analysis (DEA) framework was applied to evaluate the efficiency of national responses. Policy inputs, including healthcare resources, economic support, and stringency indices, were assessed against outputs reflecting adverse outcomes, namely COVID-19 cases, mortality, and virus transmission. Data were obtained from the Oxford COVID-19 Government Response Tracker and other publicly available international databases.

**Results:**

Marked heterogeneity in policy efficiency was observed. Countries that implemented early, stringent, and adaptive measures demonstrated superior efficiency scores. Italy, Greece, and Austria consistently ranked among the most efficient, while nations with delayed or inflexible strategies performed less effectively despite comparable resource availability. Importantly, policy flexibility and dynamic adjustment to epidemiological trends emerged as critical determinants of sustained efficiency.

**Conclusions:**

This study provides a robust comparative evaluation of COVID-19 policy efficiency across European OECD countries. The findings emphasize that timeliness of interventions, adaptability of policy measures, and judicious resource allocation were more influential than absolute resource capacity. Incorporating efficiency assessments into pandemic preparedness strategies may enhance resilience and inform evidence-based decision-making for future global health emergencies.

**Supplementary Information:**

The online version contains supplementary material available at 10.1186/s12913-026-14508-z.

## Background

The COVID-19 pandemic, characterized by its rapid global spread and profound health and economic consequences, has posed unprecedented challenges to public health systems and economies worldwide. In response, governments have implemented a range of policies aimed at curbing the spread of the virus, reducing strain on healthcare systems, and maintaining economic stability. However, the effectiveness of these policies has varied considerably across countries, prompting increased interest in evaluating and comparing the outcomes of different policies [1].

In the context of European Union (EU) and Organization for Economic Co-operation and Development (OECD) countries, the diversity of policy responses presents a valuable opportunity to assess the relative efficiency of these measures. Examining the disparities in outcomes, such as infection rates, mortality, and economic impacts, highlights the importance of understanding the factors that contribute to effective pandemic management. This evaluation is crucial for identifying the most efficient approaches to public health and economic interventions [2, 3].

The Oxford COVID-19 Government Response Tracker (OxCGRT), developed by the Blavatnik School of Government at the University of Oxford, has been a vital resource for systematically cataloging and analyzing government responses to the pandemic worldwide. OxCGRT monitors a broad range of policy measures, including containment and closure strategies, economic support, and health system initiatives. This data is compiled into indices that enable global cross-country comparisons of the stringency and comprehensiveness of governmental interventions over time. By providing a standardized set of indicators, OxCGRT serves as an indispensable tool for evaluating the impact of government actions on pandemic outcomes [4].

Studies utilizing OxCGRT data have demonstrated that stringent and timely interventions, such as lockdowns and physical distancing, were generally effective in reducing virus transmission [5, 6]. However, their effectiveness varied significantly across regions and countries, shaped by factors including demographic, economic, and geographical characteristics as well as governmental structures and levels of public compliance [7]. Comparative analyses, including those across European countries, have underscored the importance of context-specific strategies and the challenges of implementing uniform policies in diverse socio-political environments. Moreover, the OxCGRT data has been instrumental in validating non-pharmaceutical interventions (NPIs), offering a valuable framework for future pandemic preparedness and response [8]. These studies contribute to the growing body of literature that seeks to understand how varying policy approaches have influenced the pandemic’s trajectory.

Although existing studies have provided valuable insights, they often rely on descriptive and regression analyses [9], which did not fully capture the multidimensional nature of government responses and their outcomes. Regression and other econometric approaches typically focus on specific outcomes or individual policy instruments and require strong assumptions regarding functional form and variable relationships. In contrast, data envelopment analysis (DEA) offers a flexible, non-parametric framework that enables the simultaneous assessment of multiple heterogeneous inputs and outputs, including undesirable outcomes such as infections and deaths. By comparing decision-making units against best-performing peers, DEA provides a holistic evaluation of policy efficiency that reflects how governments minimize adverse health outcomes under multiple resource and policy constraints. This multidimensional benchmarking approach makes DEA particularly well suited for evaluating the overall efficiency of complex COVID-19 policy responses, enabling a more nuanced and holistic evaluation of policy efficiency [10].

Several studies have utilized DEA to compare the efficiency of COVID-19 policies across various countries and regions. A study by Mohanta et al. applied DEA to evaluate the efficiency of COVID-19 management in India, comparing states based on healthcare resources and pandemic response effectiveness [11]. Similarly, Kuzior et al. used DEA to assess the economic efficiency and resilience of public health systems in 22 countries, finding that countries following the Beveridge model were particularly effective [12]. Studies by Klumpp et al. and Selamzade et al. examined the health policy efficiency of 19 and 38 OECD countries, respectively, offering insights into the effectiveness of different governmental responses [2, 3]. These studies underscore the value of DEA in assessing the multifaceted impacts of pandemic policies by considering various inputs (e.g., healthcare resources, economic measures) and outputs (e.g., infection rates, economic stability).

Despite the valuable contributions of existing studies, a significant knowledge gap remains in the comprehensive evaluation of COVID-19 policy efficiency, particularly in a broader context. In this study, the term “comprehensive” refers to the simultaneous evaluation of multiple policy inputs and adverse health outcomes across countries and over time, using a unified analytical framework that captures both static efficiency and dynamic productivity changes. Existing studies often relied on a limited set of input variables, primarily focused healthcare resources, and were constrained by short time periods, without addressing the dynamic nature of efficiencies over time [2, 3]. There is a clear need for a more integrated approach that simultaneously considers both multiple inputs and outputs and their fluctuation, especially in a crisis as complex as COVID-19.

This study aims to address these gaps by applying DEA to evaluate the efficiency of COVID-19 policies across 27 EU OECD countries, using data from the OxCGRT to provide a comprehensive understanding of how government responses influenced the major pandemic outcomes. DEA is an ideal tool for evaluating the complex trade-offs in pandemic response because it can assess efficiency by considering multiple inputs and outputs together. By examining how multiple inputs, including demographic characteristics, policy strictness, economic support, and healthcare capacity, may minimize COVID-19-related adverse health outcomes, this study seeks to answer the following research questions:

1. How efficient were COVID-19 policy responses across 27 European OECD countries?
2. Which countries demonstrated relatively higher efficiency in managing the pandemic, and how did their performance change over time?
3. How did policy efficiency and productivity evolve throughout the pandemic?

By addressing these questions, this study aims to offer valuable insights that can guide future public health strategies and improve pandemic preparedness in Europe.

## Methods

### Study setting

This study included the 27 OECD countries in Europe as of August 22, 2024 [13]. The selection of the countries aligns with the scope of the Swedish Research Council–supported research project (award number 2022–06297) underpinning this study, which focuses on pandemic policy responses in Europe. This restriction also enhances comparability in institutional context, data availability, and policy frameworks across countries. These countries are Austria (AUT), Belgium (BEL), Czechia (CZE), Denmark (DNK), Estonia (EST), Finland (FIN), France (FRA), Germany (DEU), Greece (GRC), Hungary (HUN), Iceland (ISL), Ireland (IRL), Italy (ITA), Latvia (LVA), Lithuania (LTU), Luxembourg (LUX), Netherlands (NLD), Norway (NOR), Poland (POL), Portugal (PRT), Slovakia (SVK), Slovenia (SVN), Spain (ESP), Sweden (SWE), Switzerland (CHE), Turkey (TUR), and the United Kingdom (GBR). All country abbreviations in tables and figures follow the ISO 3-digit code system [14]. The study covered data from January 1, 2020, to December 31, 2022.

The baseline demographic characteristics of these 27 countries of 2019 are provided in Supplemental Table S1.

### Data sources

The data for this study were sourced from several publicly accessible databases. The time period of this study is from January 1, 2020 to December 31, 2022. Country-specific annual demographic data, including population, economic, and healthcare resource indicators, were obtained from the World Bank Open Data [15], Eurostat [16], and OECD Data Explorer [17]. Up-to-date country-specific daily COVID-19-related data, such as new cases, deaths, test numbers, vaccination coverage, and the reproduction number, were retrieved from Our World in Data [18]. Additionally, daily COVID-19 policy indices data were sourced from the OxCGRT. The use of these publicly available data was approved by the Swedish Ethical Review Authority (approval number: 2024-00606-01).

### Comprehensive evaluation index system

Based on previous studies and the consensus from the expert group brainstorming, the comprehensive evaluation index system for the DEA study was developed, as shown in Table 1. The input variables encompass four key aspects: government policy, human resources, financial resources, and material resources. The output variables represent adverse outcomes of the COVID-19 pandemic across three dimensions: incidence (new cases per 100,000 people), mortality (new deaths per 100,000 people), and transmission rate (effective reproduction number or Rt). The comprehensiveness of the evaluation lies in integrating diverse policy dimensions (including government response, stringency, and economic measures) and healthcare system capacity (including human, financial, and material resources), with multiple adverse health outcomes, while accounting for their evolution over time within a dynamic DEA framework.

**Table 1:** Comprehensive evaluation index system of COVID-19 policy efficiency

First-level Indicators	Second-level indicators	Third-level indicators
Input variables	Government policy	Daily healthcare system response index
		Daily stringency index
		Daily economic support index
	Human resource	Healthcare staff per 1000 people
	Financial resource	Annual healthcare expenditure per capita (in USD)
	Material resources	Hospital beds per 1000 people
		Daily new COVID-19 tests per 1000 people (7-day smoothed)
		Daily percentage of vaccinated population (%)
Output variables	COVID-19-related adverse health outcomes	Cases: daily new confirmed cases per 100,000 people (7-day smoothed)
		Deaths: Daily reported new deaths per 100,000 people (7-day smoothed)
		Virus transmission: daily effective reproduction number (Rt)

Detailed explanation of three policy indices can be found on the OxCGRT website. Briefly, the healthcare system response index summarizes the government’s response to public health measures, including testing, contact tracing, facial coverings, vaccination policies, and elderly protection. The stringency index summarizes government’s restrictions on movement and social interactions, including school and workplace closures, public event cancellations, gathering size limits, public transport closures, stay-at-home requirements, and restrictions on internal and international travel. The economic support index evaluates government measures providing income support and debt/contract relief for households. All index values have been standardized within a range of 0 to 100, where higher values indicate stronger health measures, stricter restrictions, or greater economic support [4].

### DEA method

DEA is a non-parametric method used to assess the relative efficiency of DMUs —in this study, the 27 European OECD countries—by comparing the inputs they consume (e.g. labor, capital, disease control and intervention measures) with the outputs they produce (e.g. goods, services, health outcomes). It identifies the best practices by constructing an efficient frontier, against which other DMUs are evaluated. DEA is widely applied in benchmarking, performance evaluation, and identifying opportunities for improving resource allocation and operational processes. DEA assigns varied weights derived directly from the data, optimizing the output-to-input ratio or efficiency score (*θ*) for each country relative to all others [10]. This allows each country to maximize its efficiency score by selecting the most favorable combination of weights for its inputs and outputs.

Since the origin of DEA, various types of DEA models have been developed, such as the Charnes–Cooper–Rhodes model and the Banker–Charnes–Cooper model [10]. In this study, a slack based, non-oriented super-efficiency model (SBSEM) was used to calculate the efficiency scores. Unlike input- or output-oriented models, which focus on minimizing inputs or maximizing outputs respectively, non-oriented models aim to improve both inputs and outputs simultaneously. This model is particularly useful when the goal is to achieve an optimal balance between reducing resource consumption and improving outcomes, without prioritizing one over the other. It provides a more comprehensive evaluation of efficiency, especially in complex scenarios where trade-offs between inputs and outputs are critical [19].

A super-efficiency score from SBSEM greater than one indicates that the DMU, a country, is not only efficient but also outperforms the average of other DMUs. A score of exactly one suggests that the DMU is efficient compared the average efficiency of a group that includes itself but does not exceed the performance of the group. In this case, the DMU is performing on par with the best practice frontier defined by other DMUs. A score of less than one implies inefficiency, even when the DMU is not compared against itself. This helps in identifying inefficient units. Super-efficiency is particularly useful when many or all DMUs are efficient under the traditional DEA model, as it allows for further discriminating and ranking these units. The overall efficiency may be further decomposed into technical efficiency and scale efficiency. Technical efficiency is a refined measure of overall efficiency that isolates inefficiencies due to poor resource utilization from those due to the scale of operations. It is derived from the variable returns to scale DEA, which accounts for the fact that the relationship between inputs and outputs may not always be proportional across different scales of operation. Scale efficiency refers to the ability of a DMU to operate at an optimal size or scale, meaning that it is producing at a level that maximizes productivity or efficiency [10].

Given the dynamic nature of the pandemic and the time-series structure of the data, the DEA was conducted based on a quarterly basis. For input and output variables that have daily values, their mean values of each quarter were calculated and then used in the DEA model. We also calculated the mean efficiency scores across the 12 quarters. A high mean score indicates overall efficiency throughout the study period.

We also assessed productivity change over time of the countries using the Malmquist productivity index (MPI). The MPI is widely used to evaluate improvement or regression of a DMU’s productivity across different time periods. An MPI greater than 1 indicates an increase in total productivity from one period to the next, while an MPI of 1 indicates the status quo, and an MPI less than 1 signifies a decline in productivity [10]. The MPI analysis was conducted using the same set of input and output variables as those specified in the DEA model. This ensured full consistency between the static efficiency assessment and the dynamic productivity analysis. As in the DEA framework, the output variables in the MPI analysis represent undesirable outcomes, and the same transformation approach was applied prior to analysis. Consequently, changes in productivity captured by the MPI reflect shifts in countries’ ability to utilize comparable policy inputs and resources to reduce COVID-19-related adverse health outcomes over time. MPI was further decomposed into efficiency change and technical change. Efficiency change measures whether a DMU is becoming more or less efficient over time and technical change measures whether the production frontier (representing the best possible practice) is shifting due to technological progress or regress [10].

It should be noted that the output variables included in the DEA model represent undesirable outcomes of the COVID-19 pandemic, namely new cases, new deaths, and Rt. In contrast to conventional efficiency analyses, in the present study lower values of these variables reflect improved outcomes. Accordingly, a country is considered more efficient if it achieves lower levels of COVID-19-related morbidity, mortality, and transmission while using comparable or fewer resource inputs, ceteris paribus. This treatment of undesirable outputs is well established in the DEA literature and allows efficiency to be interpreted in terms of minimizing adverse health outcomes rather than maximizing production. Following established DEA approaches for handling undesirable outputs, the undesirable variables, including stringency index, COVID-19 cases and deaths, and Rt, were transformed using a monotone decreasing function *f*(*x*) = 100 – percentile (*x*) before being included in the DEA model [20, 21]. This transformation ensures that reductions in cases, deaths, and Rt are correctly reflected as efficiency improvements in the DEA framework, thereby preserving the conceptual consistency of efficiency measurement.

All the descriptive and DEA analyses were conducted using R software version 4.41 (R Foundation for Statistical Computing, Vienna, Austria) with the *deaR* package [22].

## Results

### Country-specific characteristics of output and input variables

The distribution of the output variables included in the DEA model, measured by the medians of COVID-19 cases and deaths, and Rt, exhibited a high degree of variability across the countries. As shown in Supplemental Table S2, Slovenia reported the highest daily new cases per 100,000 people (median peak at 46.66), while Poland had the lowest (median low at 4.82). In terms of COVID-19-related mortality, Greece experienced the highest daily new deaths per 100,000 people (median peak at 0.31), whereas Iceland reported the lowest (median low at 0.01). The median of daily Rt are relatively comparable among the 27 countries ranging from 0.99 to 1.14 with the highest values seen in Sweden (1.14) and the lowest one seen in Iceland (0.99). This considerable variation underscores the diverse challenges countries encountered in managing the pandemic, particularly in terms of policy stringency and healthcare resources accessibility.

Country-specific inputs for the DEA are summarized in Supplemental Tables S3-S5. The key input variables included in the DEA model are the healthcare system response, stringency, and economic support indices (Table S3). Greece implemented the most stringent policy, reflected by high values in the healthcare system response and stringency indices, with values of 66.50 and 64.22, respectively. In contrast, Sweden’s more relaxed approach resulted in the lowest values for the two indices, with values of 37.75 and 31.23, respectively. Austria and Ireland provided the highest levels of economic support (median index value = 100), while Turkey offered the least (median index value = 11.41), which likely influenced their respective capacities to mitigate the pandemic’s impact on public health.

Denmark and Switzerland reported the highest levels of human and financial resources, with 95.04 healthcare staff per 1000 people and an annual healthcare expenditure of USD 6927 per capita, respectively. In contrast, Turkey had the lowest figures, with corresponding values of 19.07 healthcare staff and USD 1549 per capita (Table S4).

Material resources also varied significantly across countries. Germany, Austria, and the United Kingdom (UK) had relatively abundant resources, as indicated by the highest numbers of hospital beds per 1000 people, new tests per 1000 people, and percentage of vaccinated population, with median values of 8.06, 14.62, and 62.64%, respectively. These resources were crucial in supporting an effective pandemic response (Table S5).

### COVID-19 policy efficiency

**Fig. 1:**
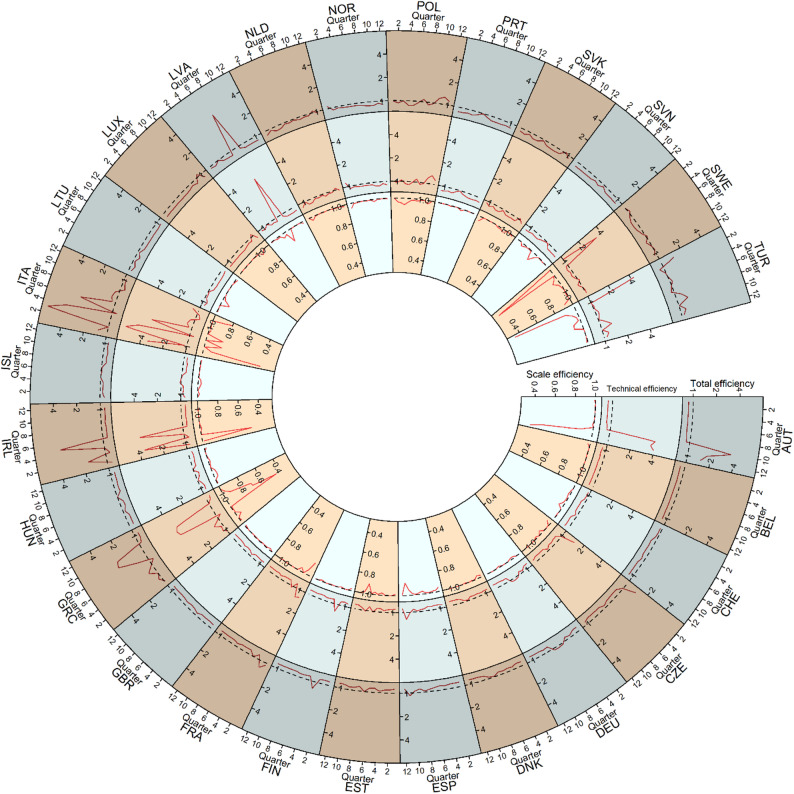
Quarterly total, technical, and scale efficiency scores of COVID-19 policies for the countries over the 3-year period

The dynamic efficiency score plot (Fig. 1) highlights that several countries, Austria, Greece, Ireland, Italy, and Latvia, exhibited notable spikes in total efficiency, reflecting periods when their COVID-19 policies outperformed the average efficiency of other countries. For example:

- Austria and Greece experienced increases in total efficiency during the later stages of the pandemic (quarters 8–12).
- Latvia showed high total efficiency in the middle of the observed period (quarter 7).
- Ireland and Italy exhibited fluctuating total efficiency scores, with periods of high and low performance during the early stages the pandemic (quarter 2–6).

In contrast, many other countries maintained relatively stable total efficiency scores throughout the pandemic, generally hovering below 1. This indicates consistent but lower total efficiency in their responses, with little evidence of significant improvement over time.

When examining technical efficiencies (Fig. 1), countries such as Austria, Greece, Ireland, Italy, Latvia, Sweden, and Turkey exhibited significant spikes, reflecting periods of notable improvement in technical efficiency. Specially, Ireland and Italy exhibited recurring spikes, reflecting fluctuating improvements during those periods. Other countries maintained low technical efficiency scores throughout the quarters, with no noticeable improvements.

Regarding scale efficiency, the results (Fig. 1) show that most countries maintained consistently high scale efficiency scores close to 1, indicating optimal scale operations throughout the pandemic. However, some countries, such as France, Ireland, Sweden, and Turkey, experienced temporary inefficiencies with significant drops around quarters 1 to 3. Austria exhibited sharp declines in scale efficiency during quarters 11 and 12, while Greece and Italy displayed recurring fluctuations, highlighting potential difficulties in sustaining optimal scale operations over time. Despite the overall stability observed in many countries, these inefficiencies underscore challenges in achieving and maintaining a stable optimal scale.

**Fig. 2:**
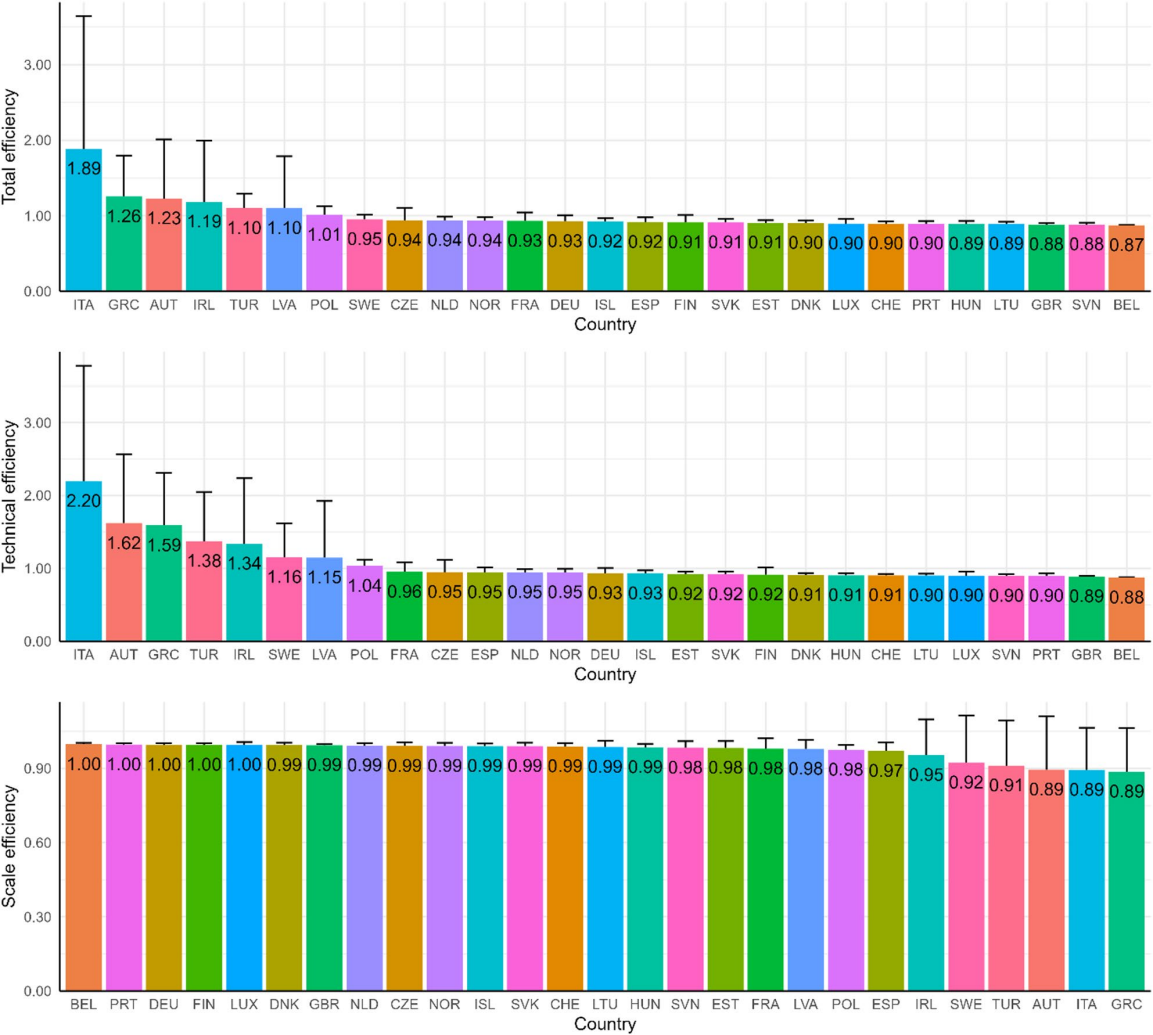
Country rankings based on efficiency scores (mean values with standard deviations of the 12 quarters)

Figure 2 presents the performance of countries based on the average values of total efficiency, technical efficiency, and scale efficiency over the 12 quarters. In total efficiency, Italy, Greece, and Austria lead with the highest scores, reflecting overall strong performance, while countries such as the UK, Slovenia, and Belgium rank lowest. For technical efficiency, Italy, Austria, and Greece exhibit the highest scores, suggesting strong operational performance, while Portugal, the UK, and Belgium are at the bottom, highlighting areas for improvement. In scale efficiency, countries such as Belgium, Portugal, Germany, Finland, and Luxembourg achieve perfect scores, reflecting optimal scale operations, whereas Austria, Italy, and Greece score the lowest, indicating suboptimal scaling. Overall, while certain countries like Italy excel in technical efficiency, they lag in scale efficiency, highlighting inefficiencies in scale despite operational strengths. Conversely, countries like Belgium excel in scale efficiency but show lower technical efficiency. High scale efficiency, but low total efficiency and/or technical efficiency means that the size of the operation is not the problem; the DMU is at the right scale, however, the inefficiency is related to how resources are managed internally i.e. technical inefficiency. In this situation, the countries should focus on internal improvements such as better resource management, cutting inefficiencies, or streamlining processes. In essence, the countries have an optimal structure or capacity, but they need better operational practices to reach full potential in terms of overall efficiency.

Although scale efficiency estimates were close to one for many countries, the descriptive statistics reveal substantial heterogeneity in both input and output variables across the 27 countries. For example, median daily COVID-19 cases ranged from fewer than 5 per 100,000 people in Poland to more than 45 in Slovenia, while median COVID-19-related mortality varied by more than thirty-fold across countries (Table S2). Policy responses also differed markedly, with healthcare system response and stringency indices substantially higher in countries such as Greece and Italy than in Sweden and Turkey (Table S3). Likewise, large disparities were observed in healthcare staffing levels, healthcare expenditure per capita, testing intensity, and vaccination coverage (Table S4 and S5). These variations indicate that countries faced markedly different pandemic conditions and resource constraints, despite operating within broadly comparable health system scales.

### COVID-19 policy productivity change

**Fig. 3:**
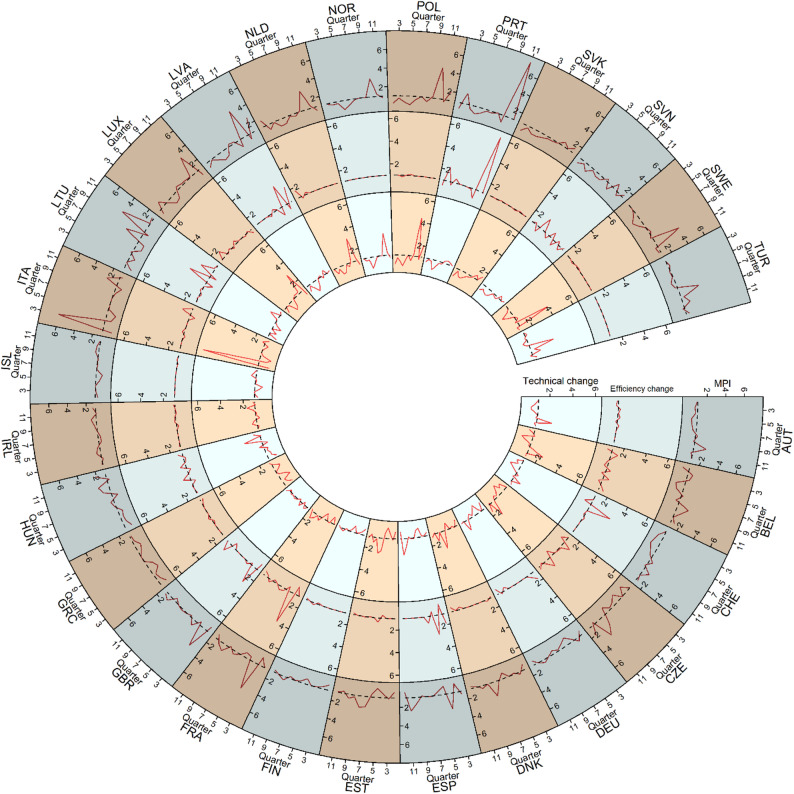
Quarterly productivity change (MPI), efficiency change, and technical change of COVID-19 policies for the countries over the 3-year period (dotted line = 1, indicating no change)

As illustrated in Fig. 3, most countries exhibit fluctuating but relatively minor productivity changes (indicated by MPI) across the quarters, with occasional spikes or dips. Notable peaks in productivity are observed in countries such as Czechia, France, the UK, Italy, Lithuania, Latvia, Poland, Portugal, and Sweden, indicating the periods of significant productivity improvements. Moderate variability is observed in nations like Austria, Belgium, Denmark, Estonia, Hungary, Luxembourg, Netherlands, Norway, and Turkey. In contrast, countries like Switzerland, Germany, Finland, Ireland, Iceland, Slovakia, and Slovenia show minimal fluctuations, reflecting consistent productivity levels throughout the period. These trends highlight differences in productivity dynamics across countries, suggesting that some experienced notable productivity growth in certain periods, while others maintained steady levels. Factors driving these patterns could include technological advancements, policy changes, or shifts in economic conditions.

The further decomposed productivity change is also shown in Fig. 3. Most countries exhibit stable efficiency change values near 1, indicating relatively constant efficiency levels over time (Fig. 3). A few countries, however, display notable upward spikes in efficiency. For instance, Switzerland, Spain, France, and the UK exhibit significant increases during the early stages of the pandemic (quarters 3–5), while Lithuania, Latvia, and Portugal demonstrate spikes in the later stages (quarters 8–12). Slovenia experiences fluctuating changes in efficiency throughout the pandemic. These peaks likely indicate catch-up processes or exceptional performance improvements during specific quarters in these countries. From a technical perspective (Fig. 3), most countries exhibit relatively minor change values close to or slightly above 1, indicating modest technological progress or stagnation. Significant spikes are observed in only few countries, such as Italy, Luxembourg, Netherlands, Norway, Poland, Sweden, and Turkey, suggesting periods of substantial technological advancement in specific quarters. Italy, for example, experiences a prominent peak early in the timeline, while Poland and Sweden exhibit a notable spike in the later period. Countries like Austria, Belgium, Switzerland, and Germany show minimal fluctuations, indicating steady or stagnant technological progress over time. Moderate variability is observed in countries like Denmark, Spain, and Estonia, where technical change values rise occasionally but remain close to the baseline. Overall, the patterns suggest that while technological progress has been steady for most countries, certain nations experienced episodic breakthroughs, likely driven by specific innovations or sectoral advancements.

**Fig. 4:**
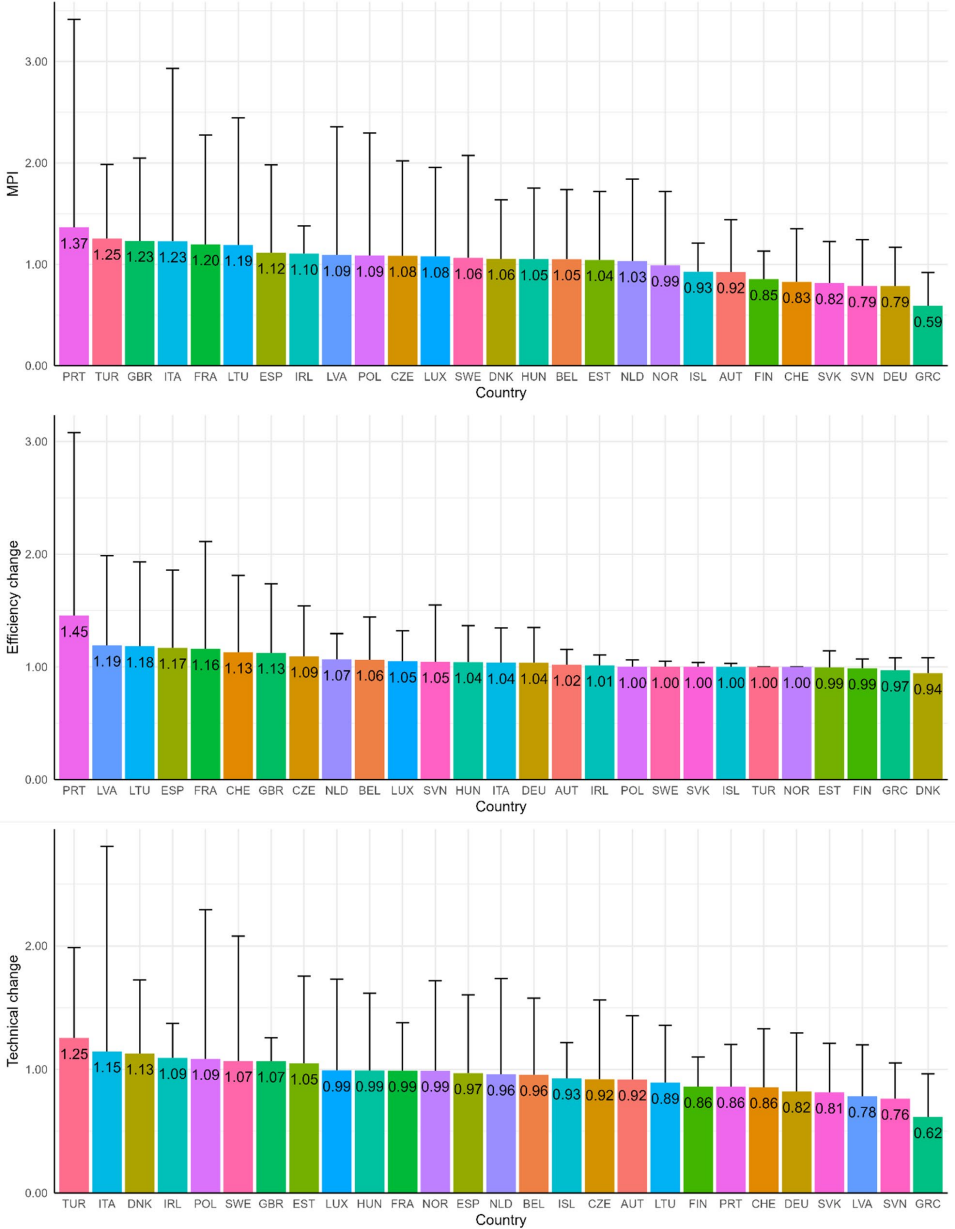
Country rankings based on productivity change scores (means values with standard deviations of quarters 2–12)

Figure 4 illustrates country rankings based on average scores across the three productivity change metrics. The MPI shows that the Portugal (1.37), the Turkey (1.25), and the UK (1.23) rank highest, indicating significant overall productivity improvements. However, significant variability in productivity change dynamics was observed in these countries, as evidenced by their large standard deviation bars. In contrast, countries like Greece (0.59), Germany (0.79), and Slovenia (0.79) exhibit the lowest MPI scores, reflecting declining productivity overall. Most countries hover around 1, signaling no or modest productivity change overall. For efficiency change, Portugal (1.45), Latvia (1.19), and Lithuania (1.18) lead, suggesting these countries made substantial gains in relative efficiency (catch-up effect). At the lower end Denmark (0.94), Greece (0.97), Estonia (0.99) and Finland (0.99) exhibit no efficiency improvements. Overall, the scores are closely clustered around 1, with relatively smaller error bars reflecting consistent performance across most countries. In technical change, Turkey (1.25), Italy (1.15), and Denmark (1.13) demonstrate the highest levels of technological progress, however, with pronounced variability, indicating uneven technological shifts over time. On the other hand, Greece (0.62), Slovenia (0.76), and Latvia (0.78) rank the lowest, reflecting limited technical advancements. Most other countries exhibit moderate or minimal changes in technical progress, with scores clustering closer to 1, suggesting relatively stable technological dynamics.

## Discussion

### Summary of findings

This study applied DEA to evaluate the efficiency of COVID-19 policy responses across 27 EU OECD countries, offering a comprehensive understanding of how governments balanced policy stringency and public health trade-offs during the pandemic. The results revealed significant disparities in efficiency and productivity dynamics in policies across the countries studied.

Countries such as Italy, Greece, and Austria emerged as leaders in total efficiency (Fig. 2), demonstrating their ability to manage the pandemic effectively while optimizing their use of healthcare resources, economic support, and policy measures. These nations maintained a favorable balance between minimizing infection rates and deaths and sustaining anti-pandemic policies. Conversely, countries like Belgium, Slovenia, and the UK consistently ranked at the lower end of efficiency scores (Fig. 1), highlighting areas for improvement in their pandemic response strategies. Notably, countries like Czechia, Hungary, and Slovakia exhibited increasing productivity trends (Fig. 3), even though their average MPI remained low (Fig. 4), reflecting their ability to adapt and improve over time. In contrast, Portugal and Turkey recorded the highest average MPI scores (Fig. 4); however, their productivity declined during the later stages of the pandemic (Fig. 3).

The concentration of scale efficiency values close to one (Fig. 2) indicates that most European OECD countries operated near an optimal system scale during the pandemic, reflecting broadly comparable population-adjusted healthcare system capacity rather than methodological bias. Consequently, cross-country differences in total efficiency are primarily driven by technical efficiency, that is, how effectively available resources and policy measures were translated into reductions in COVID-19-related adverse outcomes, rather than by differences in system scale. This finding highlights that pandemic policy effectiveness depended more on the design, timing, and implementation of interventions than on the absolute size of health system resources.

Decomposing the MPI revealed nuanced insights into how efficiency change and technical change contributed to productivity variations. Portugal and Latvia excelled in efficiency change, indicating substantial gains in relative operational efficiency, or the ability to “catch up” to best practices. On the other hand, Turkey and Italy demonstrated strong technical progress, suggesting effective adoption of innovations and advancements in their pandemic response technologies. Despite these successes, many countries exhibited considerable variability in their efficiency and technical changes over time, while most showed little to no improvement, highlighting the challenges in maintaining consistency and enhancing policy effectiveness (Fig. 4).

The study also highlighted significant fluctuations in the efficiency and productivity change metrics during different stages of the pandemic. For example, countries like Ireland and Italy displayed higher efficiency scores in the early stages, while nations such as Austria and Greece exhibited improved efficiency during the later quarters of the pandemic (Fig. 1). These variations suggest the importance of adaptability and flexibility in governmental responses to evolving circumstances.

### Comparison with previous studies

The findings of this study are consistent with prior research emphasizing the importance of stringent and timely interventions in mitigating the adverse impacts of COVID-19. For instance, previous DEA-based analyses, such as those by Klumpp et al. [3] and Kuzior et al. [12], identified wide variability in pandemic policy efficiency across countries. These studies demonstrated that countries with higher healthcare capacities and well-coordinated responses tended to perform better, a pattern that our study corroborates. However, our study contributes additional insights by adopting a dynamic approach, capturing how efficiency and productivity evolved over time rather than relying solely on static comparisons.

The strong performance of countries like Italy and Austria (Fig. 2) aligns with findings from Selamzade et al. [2], who identified these nations as effective in leveraging their healthcare resources and implementing comprehensive public health measures. The strong performance of Italy observed in our analysis is also consistent with recent causal evidence from Bonanno and De Luca, who used a difference-in-differences approach, demonstrated that post-vaccination mortality in Italy declined significantly following vaccine rollout, countering common misconceptions regarding vaccine effectiveness [23]. Our findings complement this evidence by showing that Italy’s broader policy response, encompassing healthcare system measures, vaccination, testing, and policy stringency, was efficient relative to other European OECD countries. Similarly, the high MPI scores of the UK and Italy (Fig. 4) are consistent with earlier studies suggesting that flexible and adaptive policies were crucial for sustained pandemic management. In contrast, countries like Switzerland and Slovakia (Fig. 4), which ranked lower in our analysis, reflect findings from studies such as Kuzior et al. [12], which highlighted challenges faced by nations with limited adaptability or less stringent interventions.

### Implication of the findings

Efficiency in this study should be interpreted as the ability to minimize adverse health outcomes rather than to maximize conventional outputs, reflecting the unique characteristics of pandemic policy evaluation. The efficiency of COVID-19 policy responses across the 27 countries was shaped by a combination of factors. A key factor was the timing and stringency of early interventions. Countries like Italy and Greece, which implemented strict lockdowns [24], early mass testing, and rigorous contact tracing, achieved high efficiency scores, suggesting that decisive early action helped contain the virus while optimizing resource use. In contrast, countries with delayed responses (e.g., the UK and Belgium) exhibited lower efficiency, highlighting the consequences of late-stage policy implementation.

Another critical element was policy flexibility and adaptability [25]. Countries such as Ireland and Italy, which also adjusted restrictions dynamically in response to evolving conditions, demonstrated fluctuating and high efficiency scores, suggesting that reactive policymaking may be more effective than static, long-term restrictions. In contrast, overly rigid policies may have contributed to inefficiencies in some cases, as governments struggled to adapt to changing pandemic dynamics [26].

Moreover, the balance between economic support, healthcare system response, and resource management significantly influenced policy efficiency. While Germany and Switzerland had high healthcare resources, they did not rank among the most efficient countries, suggesting that resource availability alone was not sufficient. In contrast, Austria and Ireland, despite having moderate healthcare resources and mid-level compliance with restrictions, demonstrated higher overall efficiency. This suggests that efficiency was driven not just by the quantity of resources but by how effectively they were allocated and utilized.

By incorporating these strategic insights, our findings emphasize that effective pandemic response requires a combination of early intervention, policy adaptability, and efficient resource allocation. Future policy planning should integrate these lessons to improve both immediate crisis response and long-term public health resilience.

The DEA-based findings have important implications for future crisis governance and public health system design. By identifying countries that consistently operated near the efficiency frontier, the analysis provides practical benchmarks for assessing policy performance and informing evidence-based resource allocation. The results demonstrate that comparable levels of healthcare capacity and economic support can lead to markedly different outcomes depending on policy timing, coordination, and implementation quality.

At both EU and OECD levels, comparative efficiency analysis supports cross-country learning and policy coordination by identifying transferable best practices in crisis management. Rather than emphasizing system expansion alone, the findings highlight the central role of technical efficiency, namely, effective governance, policy adaptability, and operational capacity, in building health system resilience. Integrating efficiency-based evaluations into pandemic preparedness frameworks may therefore support more resilient, coordinated, and effective responses to future public health emergencies.

### Novelty, advantages and limitations

This study contributes to the existing COVID-19 policy analysis literature in several novel ways. First, it applied a dynamic DEA framework that captures both static efficiency and productivity change over time, allowing assessment of how countries’ policy performance evolved across different stages of the pandemic. Second, by explicitly incorporating undesirable outputs, such as COVID-19 cases, deaths, and transmission intensity, the analysis aligns efficiency measurement with the core public health objective of minimizing adverse outcomes, rather than maximizing conventional outputs. Third, the focus on European OECD countries provides a policy-relevant comparative context characterized by broadly similar institutional structures, enabling clearer interpretation of efficiency differences driven by policy design and implementation rather than by system size alone. Together, these features offer new insights into pandemic governance and highlight the critical role of technical efficiency and policy adaptability in shaping effective responses.

This study also has several methodological and analytical strengths. Firstly, the use of DEA provides a robust framework for evaluating the relative efficiency, allowing for the simultaneous consideration of multiple inputs (e.g., healthcare resources, economic support, policy stringency) and outputs (e.g., infection rates, death rates, and Rt) [27]. Unlike regression-based methods, DEA does not require predefined assumptions about the relationships between inputs and outputs, making it particularly well-suited for analyzing the complex and multifaceted nature of pandemic responses [28]. Secondly, the SBSEM used in the current study offers significant advantages in evaluating complex systems like COVID-19 policy efficiency. By accounting for both input surpluses and output shortfalls, it provides a comprehensive assessment of inefficiencies. Its non-oriented nature allows for the simultaneous improvement of inputs and outputs, ensuring a balanced evaluation of resource utilization and outcomes. The super-efficiency extension enhances discriminatory power by ranking efficient DMUs beyond the standard efficiency threshold, enabling a more precise differentiation among high performers [29]. Thirdly, the dynamic nature of the analysis is another key strength, which assesses changes in efficiency and productivity over a three-year period. This approach captures the temporal variability of pandemic responses, highlighting how countries adapted their policies in response to evolving challenges [30]. Moreover, the decomposition of MPI into efficiency change and technical change offers a granular perspective on how countries achieved productivity changes [31], an aspect underexplored in previous studies. Additionally, our study incorporated undesirable outputs into the DEA framework, which enables a more nuanced evaluation of policy effectiveness, aligning with recent research advocating for the inclusion of negative outcomes in efficiency assessments [32].

However, the study is not without limitations. One notable limitation is the reliance on publicly available datasets, which may not fully capture the contextual factors influencing policy effectiveness. For example, variables such as public compliance and healthcare system resilience (though critical to understanding pandemic outcomes) were not explicitly included in the analysis [33]. Changes in standards and strategies for identifying COVID-19 cases and related deaths across countries and over time could introduce variations in data accuracy [34], thereby affecting DEA results. To ensure a fairer comparison, these issues should be further addressed in future research by incorporating additional measures for relevant policies and strategies. Additionally, the selection of input and output variables, while guided by prior research and expert consensus, could influence the results. The exclusion of certain variables, such as workforce loss and mental health outcomes [35, 36], may limit the comprehensiveness of the findings. DEA’s sensitivity to data quality and variable selection is another limitation [37]. While the method is powerful for relative efficiency comparisons, its results depend on the accuracy and completeness of the input-output data. Moreover, DEA cannot account for unobservable factors [38]. such as public trust in government or variations in healthcare quality, which may have significantly influenced pandemic outcomes.

### Directions for future research

Building on the findings and limitations of this study, several directions for future research can be proposed. First, future studies should incorporate additional contextual variables to provide a more comprehensive evaluation of policy efficiency. For instance, factors such as public compliance and vaccine distribution equity could offer valuable insights into the broader determinants of pandemic success. Including social and economic variables, such as income inequality and unemployment rates, could also help contextualize the efficiency of policy responses.

Second, subnational analyses could provide a deeper understanding of regional variations within countries, particularly in federal systems where pandemic policies may differ significantly across states or provinces. Such analyses could identify the best practices at the local level and inform more targeted interventions in future public health crises.

Third, longitudinal studies that extend beyond the acute phase of the pandemic could shed light on the long-term impacts of initial policy decisions. Examining post-pandemic recovery strategies, such as economic stimulus measures and mental health interventions [39], could provide valuable lessons for improving resilience and preparedness.

From a methodological perspective, integrating DEA with machine learning approaches could enhance the predictive capabilities of efficiency assessments. For example, machine learning algorithms could be used to identify patterns and predictors of high efficiency, enabling governments to optimize their responses in real-time [40]. Additionally, future studies could explore hybrid models that combine DEA with other analytical frameworks, such as stochastic frontier analysis or system dynamics modeling [41], to address some of the method’s limitations.

Finally, future research should consider the equity dimensions of pandemic policies. Evaluating how policies impacted vulnerable populations—such as low-income groups, minorities, and frontline workers—could provide critical insights into the social implications of pandemic management. This equity-focused perspective could guide the development of more inclusive and just public health strategies [42].

## Conclusions

This study highlights DEA as a valuable tool for assessing the efficiency of COVID-19 policy responses across 27 European OECD countries. Our findings reveal that early and stringent interventions contributed to higher efficiency, while adaptive policymaking also played a key role in sustained performance. Specifically, policies that restricted meetings, movement, and implemented widespread testing early in the pandemic were associated with more effective containment and better resource optimization. These early interventions helped reduce infection rates and relieve pressure on healthcare systems, underscoring the critical role of rapid response strategies.

Beyond policy timing, flexibility and adaptability in response measures were also crucial for long-term effectiveness. Countries that adjusted restrictions dynamically based on epidemiological trends and socioeconomic factors demonstrated more sustained efficiency over time. This adaptability allowed governments to balance public health concerns with economic stability, improving overall policy effectiveness. Conversely, nations with rigid or delayed responses faced challenges in maintaining efficiency, reinforcing the importance of agile and responsive governance in crisis situations.

Efficient resource allocation, strategic decision-making, and policy adaptability were key factors influencing pandemic response outcomes. Future research should incorporate political, social, and behavioral factors to enhance the understanding of policy effectiveness and improve preparedness for future global health crises. Integrating DEA-based efficiency benchmarking into routine policy evaluation frameworks could support more adaptive and coordinated public health responses. By learning from the successes and shortcomings of various national strategies, policymakers can develop more resilient and responsive frameworks for managing future public health emergencies.

## Supplementary Information

Below is the link to the electronic supplementary material.

Supplementary Material 1

## Data Availability

All data used in this study are publicly available. COVID-19 epidemiological data can be accessed through Our World in Data (https://ourworldindata.org/coronavirus), and policy indices are available from the Oxford COVID-19 Government Response Tracker (https://github.com/OxCGRT/covid-policy-dataset).

## Abbreviations

AUT: Austria
BEL: Belgium
CHE: Switzerland
COVID-19: Coronavirus disease 2019
CZE: Czechia
DEA: Data envelopment analysis
DEU: Germany
DMU: Decision-making unit
DNK: Denmark
ESP: Spain
EST: Estonia
EU: European Union
FIN: Finland
FRA: France
GBR: United Kingdom
GRC: Greece
HUN: Hungary
IRL: Ireland
ISL: Iceland
ISO: International Organization for Standardization
ITA: Italy
LTU: Lithuania
LUX: Luxembourg
LVA: Latvia
MPI: Malmquist productivity index
NLD: Netherlands
NOR: Norway
NPI: Non-pharmaceutical intervention
OECD: Organisation for Economic Co-operation and Development
OxCGRT: Oxford COVID-19 Government Response Tracker
POL: Poland
PRT: Portugal
Rt: Effective reproduction number
SBSEM: Slack-based, non-oriented super-efficiency model
SVK: Slovakia
SVN: Slovenia
SWE: Sweden
TUR: Turkey
UK: United Kingdom
USD: United States dollar
