# Supplementary material for: Evaluation of COVID-19 policy efficiency in 27 European OECD countries: a data envelopment analysis
Source: BMC Health Serv Res. 2026 Apr 11;26:528. doi: 10.1186/s12913-026-14508-z (PMC13085466; doi:10.1186/s12913-026-14508-z)
Supplement: Supplementary file 1 — Supplementary Material 1 [file 12913_2026_14508_MOESM1_ESM.docx]

Supplemental tables

Table S1 Demographic characteristics of the 27 European OECD countries in 2019 ^a^

| Country | Population  (Thousand) | GDP per Capita  (USD) | Land Area  (km²) |
| --- | --- | --- | --- |
| Austria (AUT) | 8,880 | 50,068 | 82,520 |
| Belgium (BEL) | 11,489 | 46,641 | 30,280 |
| Czechia (CZE) | 10,672 | 23,665 | 77,206 |
| Denmark (DNK) | 5,814 | 59,593 | 40,000 |
| Estonia (EST) | 1,327 | 23,582 | 45,750 |
| Finland (FIN) | 5,522 | 48,630 | 303,930 |
| France (FRA) | 67,388 | 40,495 | 547,557 |
| Germany (DEU) | 83,093 | 46,805 | 349,390 |
| Greece (GRC) | 10,722 | 19,144 | 128,900 |
| Hungary (HUN) | 9,771 | 16,786 | 91,260 |
| Iceland (ISL) | 361 | 68,452 | 100,830 |
| Ireland (IRL) | 4,934 | 80,848 | 68,890 |
| Italy (ITA) | 59,729 | 33,674 | 295,717 |
| Latvia (LVA) | 1,914 | 17,883 | 62,227 |
| Lithuania (LTU) | 2,794 | 19,616 | 62,620 |
| Luxembourg (LUX) | 620 | 112,726 | 2,575 |
| Netherlands (NLD) | 17,345 | 52,476 | 33,670 |
| Norway (NOR) | 5,348 | 76,431 | 365,094 |
| Poland (POL) | 37,965 | 15,700 | 306,110 |
| Portugal (PRT) | 10,286 | 23,331 | 91,606 |
| Slovakia (SVK) | 5,454 | 19,382 | 48,080 |
| Slovenia (SVN) | 2,088 | 26,042 | 20,136 |
| Spain (ESP) | 47,135 | 29,582 | 499,570 |
| Sweden (SWE) | 10,279 | 51,939 | 407,284 |
| Switzerland (CHE) | 8,575 | 84,122 | 39,510 |
| Turkey (TUR) | 82,579 | 9,215 | 769,630 |
| United Kingdom (GBR) | 66,836 | 42,663 | 241,930 |

^a^ Data source: World Bank (https://data.worldbank.org/), retrieved on August 22, 2024.

Table S2. Characteristics of output variables evaluated in the DEA model ^a^

| Country | COVID-19 cases (per 100,000 people) | COVID-19 deaths (per 100,000 people) | Effective reproduction number (Rt) |
| --- | --- | --- | --- |
| AUT | 29.28 [8.18, 77.24] | 0.13 [0.07, 0.29] | 1.08 [0.95, 1.20] |
| BEL | 20.89 [11.39, 43.08] | 0.17 [0.05, 0.27] | 1.13 [1.05, 1.21] |
| CHE | 28.58 [11.11, 44.66] | 0.05 [0.03, 0.16] | 1.12 [0.97, 1.27] |
| CZE | 18.88 [4.16, 66.91] | 0.09 [0.03, 0.49] | 1.07 [0.95, 1.18] |
| DEU | 16.15 [5.02, 58.97] | 0.13 [0.09, 0.25] | 1.07 [0.98, 1.20] |
| DNK | 16.11 [9.40, 24.44] | 0.10 [0.02, 0.14] | 1.03 [0.96, 1.17] |
| ESP | 20.22 [10.01, 27.08] | 0.13 [0.10, 0.34] | 1.08 [1.03, 1.23] |
| EST | 17.89 [7.19, 33.82] | 0.12 [0.07, 0.32] | 1.06 [1.01, 1.22] |
| FIN | 9.05 [2.70, 30.58] | 0.06 [0.03, 0.25] | 1.07 [0.92, 1.14] |
| FRA | 30.90 [15.52, 69.62] | 0.12 [0.09, 0.30] | 1.05 [1.00, 1.13] |
| GBR | 23.25 [5.89, 38.05] | 0.15 [0.10, 0.31] | 1.04 [0.95, 1.13] |
| GRC | 20.65 [9.74, 57.85] | 0.31 [0.15, 0.46] | 1.06 [0.95, 1.12] |
| HUN | 14.01 [1.73, 32.01] | 0.13 [0.05, 0.88] | 1.10 [0.96, 1.27] |
| IRL | 13.40 [5.74, 33.77] | 0.10 [0.05, 0.17] | 1.07 [0.95, 1.13] |
| ISL | 9.89 [2.65, 38.54] | 0.01 [0.00, 0.06] | 0.99 [0.88, 1.17] |
| ITA | 22.12 [6.24, 56.87] | 0.17 [0.13, 0.44] | 1.05 [0.99, 1.17] |
| LTU | 26.93 [13.03, 37.45] | 0.17 [0.05, 0.59] | 1.04 [0.90, 1.20] |
| LUX | 32.69 [11.82, 60.76] | 0.09 [0.05, 0.18] | 1.08 [1.00, 1.18] |
| LVA | 24.73 [7.42, 42.34] | 0.21 [0.09, 0.49] | 1.07 [0.93, 1.20] |
| NLD | 23.57 [7.76, 32.97] | 0.06 [0.02, 0.20] | 1.08 [0.96, 1.16] |
| NOR | 7.23 [2.07, 10.35] | 0.05 [0.03, 0.13] | 1.07 [0.90, 1.14] |
| POL | 4.82 [1.24, 27.09] | 0.04 [0.03, 0.60] | 1.10 [0.93, 1.18] |
| PRT | 21.95 [5.82, 40.75] | 0.10 [0.07, 0.27] | 1.05 [0.96, 1.10] |
| SVK | 8.21 [2.54, 31.78] | 0.12 [0.01, 0.58] | 1.02 [0.86, 1.23] |
| SVN | 46.66 [12.39, 67.73] | 0.22 [0.06, 0.63] | 1.06 [0.97, 1.21] |
| SWE | 7.98 [5.48, 32.62] | 0.12 [0.05, 0.33] | 1.14 [1.03, 1.21] |
| TUR | 15.43 [4.04, 24.33] | 0.10 [0.03, 0.18] | 1.01 [0.80, 1.07] |

^a^ Median and interquartile ranges were provided for quarterly means of daily values.

Table S3. Characteristics of COVID-19 policy indices ^a^

| Country | Healthcare system response | Stringency | Economic support |
| --- | --- | --- | --- |
| AUT | 61.79 [54.76, 64.67] | 48.07 [36.56, 64.40] | 100.00 [94.23, 100.00] |
| BEL | 54.68 [37.67, 61.93] | 40.57 [18.64, 57.53] | 69.43 [0.00, 75.00] |
| CHE | 51.82 [21.34, 57.16] | 43.64 [15.96, 50.67] | 37.50 [25.36, 39.34] |
| CZE | 51.16 [33.19, 58.17] | 38.77 [23.20, 54.05] | 57.14 [31.49, 64.06] |
| DEU | 57.32 [40.04, 62.96] | 46.76 [19.02, 65.06] | 37.50 [37.50, 85.42] |
| DNK | 47.60 [21.43, 53.48] | 30.57 [16.50, 55.19] | 18.60 [0.00, 60.37] |
| ESP | 52.92 [39.05, 60.02] | 44.61 [27.14, 64.20] | 87.50 [30.67, 87.50] |
| EST | 38.57 [31.54, 48.43] | 33.92 [18.84, 44.13] | 56.67 [20.12, 72.84] |
| FIN | 42.99 [35.66, 49.49] | 31.32 [20.19, 44.93] | 66.07 [50.00, 75.00] |
| FRA | 53.92 [36.40, 64.48] | 42.33 [23.76, 62.61] | 33.42 [13.19, 50.61] |
| GBR | 51.91 [26.67, 62.61] | 43.86 [15.78, 67.04] | 62.50 [17.17, 100.00] |
| GRC | 66.50 [40.39, 74.56] | 64.22 [30.61, 70.50] | 87.50 [85.19, 98.12] |
| HUN | 44.21 [24.89, 56.96] | 29.69 [15.20, 59.66] | 36.96 [20.67, 69.45] |
| IRL | 56.78 [25.14, 67.25] | 41.74 [14.24, 67.25] | 100.00 [52.27, 100.00] |
| ISL | 43.63 [19.05, 48.94] | 31.73 [16.84, 44.19] | 89.55 [61.40, 100.00] |
| ITA | 65.75 [47.59, 72.91] | 54.24 [34.89, 74.20] | 75.00 [47.83, 75.00] |
| LTU | 45.25 [23.37, 57.93] | 32.68 [16.57, 51.86] | 71.86 [69.09, 100.00] |
| LUX | 54.93 [40.79, 58.50] | 41.17 [19.12, 48.30] | 24.18 [12.29, 68.48] |
| LVA | 46.01 [25.31, 48.68] | 41.74 [13.80, 49.43] | 38.90 [10.92, 70.39] |
| NLD | 54.03 [35.60, 60.31] | 43.42 [16.27, 65.68] | 52.58 [50.00, 65.62] |
| NOR | 40.36 [23.21, 48.67] | 33.76 [17.54, 51.11] | 37.50 [37.50, 37.50] |
| POL | 45.33 [22.19, 60.89] | 37.77 [16.17, 63.41] | 54.18 [2.88, 75.00] |
| PRT | 57.46 [30.26, 63.26] | 48.82 [18.21, 65.60] | 75.00 [0.00, 75.00] |
| SVK | 49.04 [29.55, 64.96] | 39.17 [17.21, 60.81] | 87.50 [80.60, 89.20] |
| SVN | 50.77 [26.19, 60.89] | 45.22 [14.92, 56.50] | 53.53 [5.77, 74.21] |
| SWE | 37.75 [18.98, 54.44] | 31.23 [13.12, 60.94] | 31.25 [6.49, 42.01] |
| TUR | 42.08 [25.20, 65.16] | 37.18 [18.81, 70.16] | 11.41 [0.00, 61.31] |

^a^ Medians and interquartile ranges were provided for quarterly means of daily values.

Table S4. Characteristics of human and financial resources ^a^

| Country | Healthcare staff per 1000 people | Annual healthcare expenditure per capita (in USD) |
| --- | --- | --- |
| AUT | 55.17 | 5683.87 |
| BEL | 57.54 | 5198.93 |
| CHE | 84.81 | 6927.36 |
| CZE | 35.83 | 3559.4 |
| DEU | 75.4 | 6448.2 |
| DNK | 95.04 | 5102.31 |
| ESP | 35.01 | 3632.97 |
| EST | 31.09 | 2557.04 |
| FIN | 81.75 | 4644.61 |
| FRA | 59.63 | 5234.48 |
| GBR | 61.85 | 4551.59 |
| GRC | 26.8 | 2494.88 |
| HUN | 33.31 | 2393.19 |
| IRL | 61.28 | 5353.01 |
| ISL | 69.44 | 4387.17 |
| ITA | 34.14 | 3326.22 |
| LTU | 35.69 | 2824.84 |
| LUX | 84.06 | 5545.78 |
| LVA | 28.11 | 2756.11 |
| NLD | 89.94 | 5495.53 |
| NOR | 111.6 | 6210.47 |
| POL | 28.76 | 2339.77 |
| PRT | 41.41 | 3466.61 |
| SVK | 26.34 | 2581.43 |
| SVN | 34.74 | 3286.08 |
| SWE | 82.47 | 5309.83 |
| TUR | 19.07 | 1549.98 |

^a^ Medians were provided for annual values.

Table S5. Characteristics of material resources

| Country | Hospital beds per 1000 people ^a^ | New tests per 1000 people ^b^ | Percentage of vaccinated population (%) ^b^ |
| --- | --- | --- | --- |
| AUT | 7.37 | 14.62 [2.31, 38.9] | 47.48 [0.36, 76.48] |
| BEL | 5.62 | 2.64 [1.09, 4.12] | 52.93 [0.00, 78.75] |
| CHE | 4.65 | 2.11 [1.03, 3.02] | 42.76 [0.00, 69.35] |
| CZE | 6.63 | 2.10 [0.35, 8.68] | 42.30 [0.01, 66.09] |
| DEU | 8.06 | 1.51 [1.06, 2.10] | 49.89 [0.03, 77.03] |
| DNK | 2.60 | 8.01 [1.43, 20.67] | 48.95 [0.00, 80.45] |
| ESP | 2.97 | 1.89 [0.92, 2.93] | 51.42 [0.17, 86.06] |
| EST | 4.69 | 2.27 [0.88, 3.46] | 40.66 [0.01, 64.56] |
| FIN | 3.61 | 2.18 [0.72, 3.01] | 52.72 [0.00, 79.96] |
| FRA | 5.98 | 3.63 [2.67, 5.57] | 49.16 [0.00, 79.95] |
| GBR | 2.50 | 4.58 [3.48, 13.85] | 62.64 [3.39, 78.01] |
| GRC | 4.20 | 6.73 [1.83, 10.12] | 42.23 [0.00, 75.19] |
| HUN | 7.01 | 0.73 [0.45, 1.89] | 50.51 [1.30, 63.96] |
| IRL | 2.97 | 2.08 [1.04, 3.76] | 54.92 [36.37, 80.87] |
| ISL | 2.87 | 2.27 [1.40, 4.07] | 56.28 [1.31, 82.23] |
| ITA | 3.17 | 3.51 [2.35, 4.43] | 51.25 [0.01, 85.40] |
| LTU | 6.56 | 2.27 [0.89, 5.15] | 34.24 [0.00, 67.84] |
| LUX | 4.51 | 5.49 [2.64, 11.22] | 45.80 [0.07, 73.74] |
| LVA | 5.57 | 2.40 [1.00, 6.37] | 31.39 [0.00, 72.34] |
| NLD | 3.28 | 1.28 [0.28, 2.59] | 46.72 [0.20, 71.25] |
| NOR | 3.60 | 2.37 [0.33, 3.24] | 48.37 [0.00, 79.63] |
| POL | 6.62 | 0.76 [0.20, 1.50] | 37.39 [0.01, 56.27] |
| PRT | 3.39 | 4.44 [2.74, 4.96] | 53.95 [0.05, 94.39] |
| SVK | 5.78 | 2.60 [0.40, 8.65] | 33.49 [0.53, 45.80] |
| SVN | 4.49 | 1.52 [1.02, 2.34] | 36.74 [0.30, 59.55] |
| SWE | 2.21 | 1.49 [0.23, 3.11] | 44.17 [0.01, 72.99] |
| TUR | 2.81 | 1.77 [1.41, 2.97] | 36.23 [3.59, 67.48] |

^a^ Medians were provided for annual values.

^b^ Medians and interquartile ranges were provided for quarterly means of daily values.
